# Preventive Effects of the Intestine Function Recovery Decoction, a Traditional Chinese Medicine, on Postoperative Intra-Abdominal Adhesion Formation in a Rat Model

**DOI:** 10.1155/2016/1621894

**Published:** 2016-12-26

**Authors:** Cancan Zhou, Pengbo Jia, Zhengdong Jiang, Ke Chen, Guanghui Wang, Kang Wang, Guangbing Wei, Xuqi Li

**Affiliations:** ^1^Department of Hepatobiliary Surgery, The First Affiliated Hospital of Xi'an Jiaotong University, Xi'an, Shaanxi 710061, China; ^2^Department of General Surgery, The First People's Hospital of Xianyang City, Xianyang, Shaanxi 712000, China; ^3^Department of General Surgery, The First Affiliated Hospital of Xi'an Jiaotong University, Xi'an, Shaanxi 710061, China

## Abstract

The intestine function recovery decoction (IFRD) is a traditional Chinese medicine that has been used for the treatment of adhesive intestinal obstruction. In this study, the preventative effects and probable mechanism of the IFRD were investigated in a rat model. We randomly assigned rats to five groups: normal, model, control, low dose IFRD, and high dose IFRD. In the animal model, the caecum wall and parietal peritoneum were abraded to induce intra-abdominal adhesion formation. Seven days after surgery, adhesion scores were assessed using a visual scoring system, and histopathological samples were examined. The levels of serum interleukin-6 (IL-6) and transforming growth factor beta-1 (TGF-*β*1) were analysed by an enzyme-linked immunosorbent assay (ELISA). The results showed that a high dose of IFRD reduced the grade of intra-abdominal adhesion in rats. Furthermore, the grades of inflammation, fibrosis, and neovascularization in the high dose IFRD group were significantly lower than those in the control group. The results indicate that the IFRD can prevent intra-abdominal adhesion formation in a rat model. These data suggest that the IFRD may be an effective antiadhesion agent.

## 1. Introduction

Intra-abdominal adhesions are a common complication that occurs in 90–95% of patients who undergo abdominal surgery [1, 2]. Intra-abdominal adhesion can cause abdominal and pelvic pain, adhesive intestinal obstruction, infertility, and other severe complications [3]. Approximately 10% of bowel obstructions caused by adhesions require surgery to release the adhesions, with resulting mortality rates of 5–20% [4]. Operative adhesiolysis results in increased surgical difficulty, prolonged operative duration, and increased risk of bleeding. The situation is even more complicated because approximately 30% of patients require reoperation for adhesion recurrence [5].

The recurrence rate of adhesive bowel obstruction after treatment using a surgical method is high. Without effective precautions, the recurrence rate is 12% within 41 months of the initial surgery. The risk of relapse is present even after 20 years [6]. Adhesive bowel obstruction causes endless pain in patients and places a considerable burden on already overtaxed healthcare systems [7]. To date, there is no effective method for preventing intra-abdominal adhesion [8]. Therefore, finding an effective agent or strategy to prevent intra-abdominal adhesion is critical [9].

In China and other parts of East Asia, traditional Chinese medicine (TCM) has been used to treat disease for thousands of years. TCM usually works by mixing different types of herbs, which are called formulas or “Fufang.” As a complementary treatment, TCM may offer an option for prevention of intra-abdominal adhesion formation. According to TCM theory, intestine function recovery decoction (IFRD) has been used clinically with substantial benefits in treating adhesive intestinal obstruction. However, there is no in vivo experimental evidence showing this effect or likely pharmacological mechanisms of adhesion prevention. In this study, we demonstrate the effects of the IFRD and explain a likely mechanism for intra-abdominal adhesion prevention in a rat model.

## 2. Materials and Methods

### 2.1. Preparation of the IFRD

The constituents of the IFRD are shown in Table 1. All of the TCM herbs were purchased from the pharmacy of the First Affiliated Hospital of Xi'an Jiaotong University. Each herb was identified and authenticated by the head of the department and herbal medicinal botanist. Per the Pharmacopoeia of China (version 2010), exact amounts of component herbs were weighed according to the classic percentages and mixed well. The mixture was soaked in distilled water for 30 min and then boiled in eight volumes of water (v/w) for 1 h in herb-extracting machine. This preparation followed the ancient method and was also identical to the clinical preparation. The supernatant was orally administered to the rats in the experiments. The concentration of the IFRD is expressed as the total dry weight of the crude herbs per millilitre in decoction.

### 2.2. Operation and Postoperative Intervention

Sprague–Dawley rats weighing 200–250 g were purchased from the Experimental Animal Centre of Xi'an Jiaotong University (SYXK2012-003). All the rats were fed at room temperature (22 ± 2°C). They were allowed to drink freely and were provided with standard rat chow. All the procedures were authorized by the Animal Ethics Committee of Xi'an Jiaotong University (XJTULAC2016-410). The animals were randomly divided into five groups: normal, model, control, low dose, and high dose IFRD groups. All the rats were anesthetized using methoxyflurane prior to surgery. The skin was shaved and sterilized using iodine solution. As previously described [10], a 2 to 3 cm incision was made. The caecum wall and corresponding parietal peritoneum were scrubbed with sterile gauze until punctate haemorrhage occurred. The area of the injured wall of the caecum was approximately 2-3 cm^2^. After exposure to air for 5 min, the bowel loop was arranged and returned to its original position. In the normal group, the caecum wall and corresponding parietal peritoneum were not scrubbed.

The IFRD was administered to the rats twice a day via gastric infusion from postoperative day 0 to day 7 with 10 mL/kg/day solution. In the high dose IFRD group, the dosage is equivalent to 15.1 g of crude herbs/kg/day, which was calculated from the body surface area and is generally used clinically for humans [11]. In the low dose IFRD group, the dosage per rat is 7.55 g of crude herb/kg/day, which is equal to approximately half of the human dose. For rats in the control group, normal saline was administered at the same volume for rats in the IFRD groups. Each rat was monitored for changes in body weight. Every step of the operation and the postoperative interventions for each group are shown in Table 2.

### 2.3. Assessment of Adhesion Grade

Seven days after the operation, all the rats were anesthetized, and the abdominal cavities were opened with U-like incisions. The intra-abdominal adhesion evaluation was performed by a single researcher blinded to the treatment data according to the standard adhesion grades by Nair et al. [12]. These standard grades are listed in Table 3.

### 2.4. Haematoxylin and Eosin (HE) Staining and Microscopic Histological Grading

The injured peritoneum and adhesion tissues were excised after assessment of the adhesion grade. Specimen fixation and section preparation were carried out and then HE staining was performed. The tissues were evaluated under a microscope as previously reported [13]. The evaluation standards were as follows: degree of inflammation (grade 0: absent or normal in number; grade 1: slight increase; grade 2: moderate infiltration; and grade 3: massive infiltration), fibrosis (grade 0: none; grade 1: slight; grade 2: moderate; and grade 3: dense), and neovascularization (grade 0: none; grade 1: 1-2 vessels; grade 2: 3–9 vessels; and grade 3: 10 or more vessels).

### 2.5. Immunohistochemistry

The samples were fixed with 4% paraformaldehyde and then embedded in paraffin. After cutting the paraffin-embedded samples into sections, immunohistochemical staining was performed. The expression level of *α*-SMA (sc-53015, 1 : 800 dilution; Santa Cruz Biotechnology, Dallas, TX, USA) was detected. An image signal acquisition and analysis system (Leica, Wetzlar, Germany) was used for image acquisition.

### 2.6. Enzyme-Linked Immunosorbent Assay (ELISA)

Seven days after surgery, blood samples from the rats were obtained. After centrifugation at 3,000 rpm for 30 min, only the serum was retained and stored at −20°C. The serum levels of TNF-*α* and IL-6 were examined using the ELISA kit (R&D, Minneapolis, MN, USA) according to the manufacturer's recommendations.

### 2.7. Statistical Analysis

All the data are expressed as the mean ± standard error or the median. A one-way ANOVA was performed followed by the* LSD* method for multiple comparisons to compare differences between the groups. A Kruskal-Wallis analysis was applied for assessing the adhesion grades. A Mann–Whitney *U* analysis was used to compare intergroup differences. The statistical analysis was performed using SPSS software version 13.0. *P* < 0.05 was considered to be statistically significant.

## 3. Results

### 3.1. The IFRD Reduced the Macroscopic Grades of Intra-Abdominal Adhesion in Rat Models

**Figure 1:**
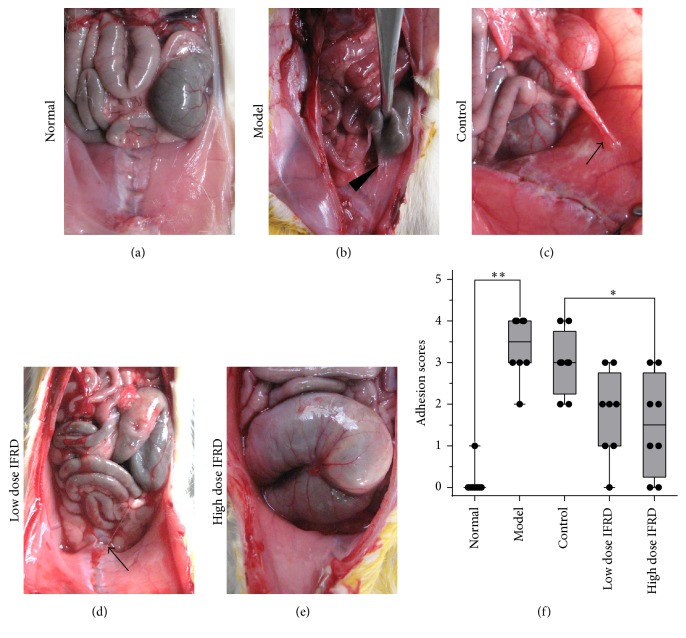
The intestine function recovery decoction (IFRD) prevented intra-abdominal adhesion formation in rats. (a) Normal group animals had intra-abdominal adhesions. (b) Model group animals developed a large number of extensive, thick adhesions, which were difficult to separate (black triangle). (c) Intra-abdominal adhesions occurred only slightly less in control group animals (black arrow). (d) The low dose IFRD group exhibited fewer intra-abdominal adhesions (black arrow) than the control group. (e) In the high dose IFRD group, some animals had no intra-abdominal adhesions. (f) Adhesion scores for the macroscopic classification (*n* = 8). The IFRD groups had the lowest scores of adhesions (^*∗*^*P* < 0.05; ^*∗∗*^*P* < 0.01).

None of the rats died and all completed the experiment. The grades of adhesion showed significant differences between the different groups. There was almost no adhesion in the normal group (Figure 1(a)), whereas the rats in the model group (Figure 1(b)) and in the control group (Figure 1(c)) demonstrated patchy adhesion that could not be separated; the adhesion appeared at the injured areas of the peritoneum and caecum surface as well as the omentum. In contrast, the adhesions in the animals of the low dose IFRD group appeared to be loose and easy to separate (Figure 1(d)). The rats in the high dose IFRD group showed slight adhesions or even no adhesions. After grading the adhesions, the five groups showed significant differences (*P* < 0.05) (Table 4 and Figure 1(e)). There was no obvious difference in the adhesion grades between the model group and the control group, excluding the possibility of gastric infusion with an equal volume of saline for adhesion prevention. Compared to the control group, we found that the IFRD, especially the high dose of IFRD, significantly reduced the grades of adhesion. Thus, the results showed that the IFRD was able to prevent intra-abdominal adhesion in rats.

### 3.2. The IFRD Inhibited Inflammation, Fibrosis, and Neovascularization in Rat Models

**Figure 2:**
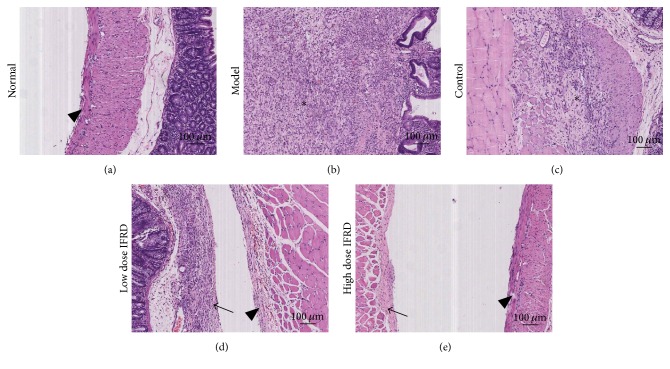
Representative images of HE staining in each group (100x). (a) Normal group; (b) model group; (c) control group; (d) low dose IFRD group; and (e) high dose IFRD group (*∗* indicates the area of adhesion tissue; black triangle indicates visceral peritoneum; black arrow indicates parietal peritoneum).

**Figure 3:**
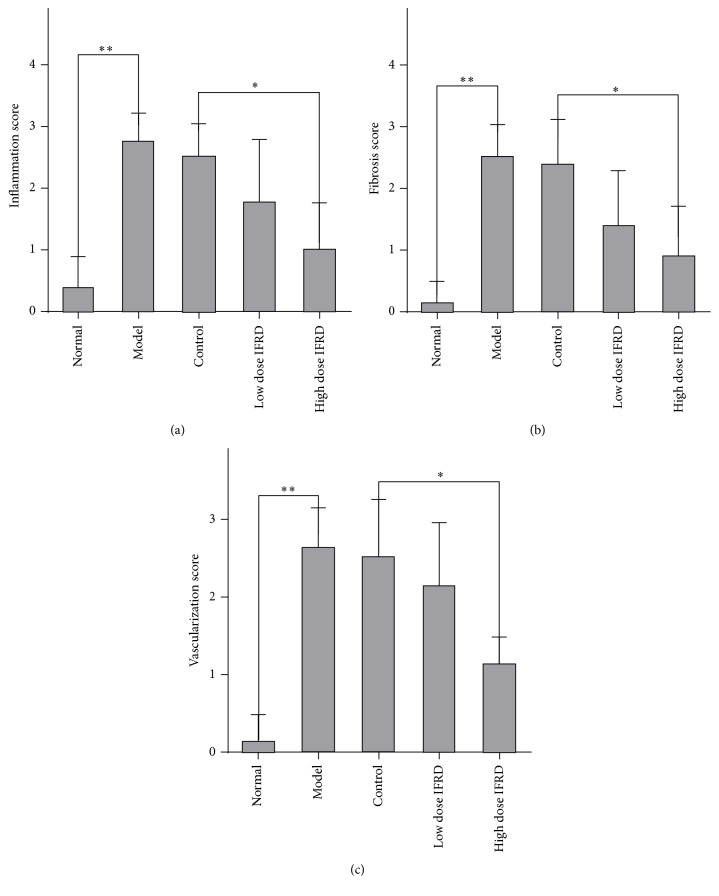
Inflammation, fibrosis, and neovascularization scores for each group (^*∗*^*P* < 0.05; ^*∗∗*^*P* < 0.01). (a) Inflammation scores, (b) fibrosis scores, and (c) neovascularization scores are shown.

By assessing the HE staining of the adhesion and peritoneum wound tissue slices, the grades of inflammation, fibrosis, and neovascularization were observed (Figure 2). We found that compared with the normal group the grades of inflammation, fibrosis, and neovascularization in the model and control groups were improved. However, compared to the control group, the high dose IFRD group distinctly presented less inflammation, fibrosis, and neovascularization (Figure 3). There is a trend to reduce inflammation, fibrosis, or neovascularization in the low dose IFRD group compared with the control group. Therefore, the data indicate that the IFRD inhibited inflammation, fibrosis, and neovascularization in the progression of adhesion induced by abrasion.

### 3.3. The IFRD Inhibited *α*-SMA, an Activated Fibroblast Marker, in Adhesion Tissues in the Rat Model

**Figure 4:**
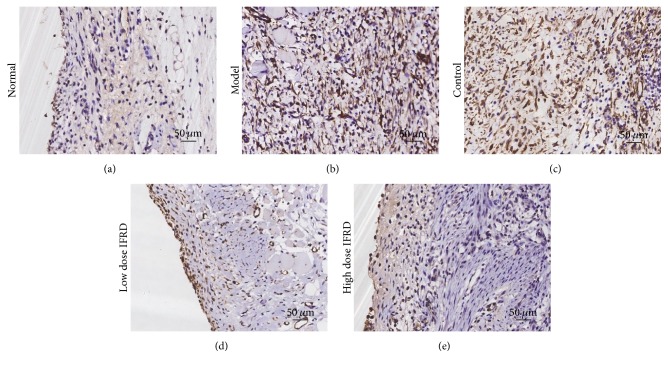
Immunohistochemical analysis of *α*-SMA in intra-abdominal adhesion tissues from all groups (200x). (a) Normal group; (b) model group; (c) control group; (d) low dose IFRD group; and (e) high dose IFRD group.

To further study the degree of fibrosis of adhesion tissues in the rat model, immunohistochemical staining of *α*-SMA, an activated fibroblast marker, was performed. In the normal group, no positive staining in the intact peritoneum was observed. In the model and control groups, there were a large number of fusiform fibroblasts with positive brown staining in the thick adhesive tissue. However, in the high dose IFRD group, the expression of *α*-SMA in the injured peritoneum and/or adhesion tissues was remarkably decreased compared to the model and control groups (Figure 4).

### 3.4. The IFRD Suppressed the Blood Levels of TGF-*β* and IL-6 in the Rat Model

**Figure 5:**
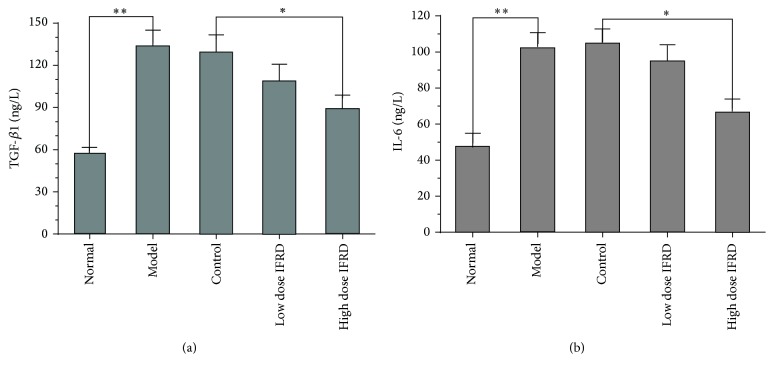
Effects of the IFRD on the serum levels of TGF-*β*1 (a) and IL-6 (b) (^*∗*^*P* < 0.05; ^*∗∗*^*P* < 0.01).

We used ELISA to analyse the levels of TGF-*β*1 and IL-6 in the serum. The results indicate that the serum levels of TGF-*β* (Figure 5(a)) and IL-6 (Figure 5(b)) were notably higher in the model and control groups than that in the normal group, suggesting that operative injury-induced adhesion formation was accompanied by significant inflammatory response. A high dose of IFRD can markedly inhibit the increase of TGF-*β* and IL-6 (*P* < 0.05).

## 4. Discussion

Intra-abdominal adhesions are a common complication after abdominal and pelvic surgery [5]. Our study showed that the TCM IFRD could effectively prevent postoperative intra-abdominal adhesions in a rat model, likely resulting from the inhibition of inflammation, fibrosis, and neovascularization during adhesion formation.

An abdominal adhesion forms between two wound surfaces of the peritoneum. The formation of an adhesion depends on whether the deposited fibrous tissue is absorbed or undergoes organization [14]. The factors resulting in adhesion formation include peritoneal mechanical trauma and peritoneal ischaemia caused by operation, traction, or residue from foreign matter, such as a suture. All these factors cause damage to the peritoneum and an inflammatory response. The cytokines released by inflammatory cells and oxidative stress are considered triggers and important initial events. The inflammatory reaction caused by peritoneal damage will produce fibrous exudation and deposition; meanwhile, the dissolving capacity of wound tissue will decrease. Ultimately, the deposition of extracellular matrix results in the formation of an adhesion [15, 16] Thus, the formation of an adhesion is a complex process caused by different cells, inflammatory mediators, and cytokines.

With the mechanism of adhesion formation elucidated, many preventative methods have emerged. Several barrier materials have been used clinically, including hyaluronic acid, carboxymethyl chitin, and oxidized regenerated cellulose, to prevent adhesion by separating the wounded tissues and promoting the repair of mesothelium cells [17–19]. Furthermore, researchers have attempted to prevent postoperative adhesion by inhibiting the inflammatory response [20], regulating fibrinolysis [21], and using antiangiogenesis [22] and antifibrosis [23] methods. However, verification of the value, effectiveness, and safety of these applications requires clinical trials and evidence-based medicine.

The IFRD consists of nine different herbs that have complex chemical components. According to the TCM theory [24, 25], the IFRD plays an important role in the special therapeutic method of “removing stasis by purgation and promoting blood circulation to remove blood stasis.” The IFRD works effectively in treating severe abnormal infection and bowel motility dysfunction. In essence, the IFRD promotes tissue repair, decreases the inflammatory reaction and exudation, improves circulation in the intestine, and eventually improves intestinal functional recovery. Our study shows that the grades of intra-abdominal adhesion were reduced and the nonadhesion percentage was elevated in rats given the IFRD. Consequently, the IFRD may have an advantage in potentially preventing intra-abdominal adhesion.

Studies have demonstrated that inhibiting the inflammatory reaction can prevent intra-abdominal adhesion in animals [26, 27]. Thus, it may be effective in preventing adhesion by inhibiting the inflammatory reaction and cytokines induction caused by injury. In the IFRD, some constituents including Codonopsis Radix (Dangshen) [28], Atractylodes Rhizoma (Baizhu) [29], and Paeoniae Rubra Radix (Chishao) [30] were reported to have anti-inflammatory and antioxidative effects. Jia and He [31] indicated that paeoniflorin, a chemical constituent of Paeoniae Rubra Radix, ameliorated disease in rat models of rheumatoid arthritis by suppressing oxidative stress and inflammation and reducing COX-2 protein expression. Wei et al. [32] and other researchers [20, 33] have provided evidence that hypoxia-induced COX-2 expression in peritoneal fibroblasts is involved in the formation of intra-abdominal adhesions. In the current study, the grade of inflammation in the IFRD group was lower than that in the control group, which demonstrates that the IFRD possesses potent anti-inflammatory properties.

Cytokines such as IL-6 and TGF-*β* are of vital importance during the formation of adhesions. IL-6 is a key cytokine for regulating the proliferation of epithelial cells and promoting the deposition of inflammatory cells and fibrosis at damage sites [34]. TGF-*β* promotes peritoneal mesothelial cells to increase the synthesis of plasminogen activator inhibitor-1 by regulating the activity of various cytokines [35] and accelerates the migration and proliferation of adhesion fibroblasts [23]. The expression levels of IL-6 and TGF-*β* positively regulate the formation of adhesions. Our present study suggests that the preventative effects of the IFRD on intra-abdominal adhesion formation are involved in inhibition of inflammatory cytokines IL-6 and TGF-*β* release.

Because the overexpression of cytokines induced by inflammation correlates with hyperplasia of fibrous connective tissue, the IFRD may work by inhibiting the expression of cytokines and decreasing hyperplasia of fibrous connective tissue. Fibroblast cells are crucial for ECM deposition; once activated, they differentiate into myofibroblasts that express *α*-SMA [30]. A hallmark of myofibroblast activation, *α*-SMA, is typically used to assess the level of fibrosis [36], and positive staining of *α*-SMA reveals the development of fibrosis. In our results, the observation of decreased fibrosis grades and *α*-SMA expression in the IFRD group demonstrated that the IFRD notably inhibited fibrosis and activated fibroblasts in adhesion formation.

Operative injury damages the balance between the production and degradation of fibrous protein while inducing the deposition of ECM, which forms the foundation of the adhesion. Persicae Semen (Taoren) in the IFRD is a representative herb for invigorating blood circulation and eliminating stasis; after thousands of years of clinical application and observation in China, its therapeutic effects are indeed certain, and its pharmacoactivity is defined [37]. Persicae Semen (Taoren) significantly decreased fibrinogen content, prolonged thrombin time and thromboplastin time, and increased prothrombin time in an animal model [38]. Therefore, the mechanism of action of the IFRD for adhesion prevention may be related to activating the fibrinolytic system and decreasing the deposition of fibrous protein.

An intra-abdominal adhesion may have restricted motility in the slow postoperative recovery of intestinal function caused by injury from the abdominal operation and anesthetization [39]. Inhibition of postoperative intestinal motility by inhibitors of gastrointestinal motility can result in increased numbers of adhesions. Therefore, the possibility of preventing postoperative adhesions by promoting gastrointestinal transit has been suggested [40]. Bove and Chapelle [41] have shown that visceral massage immediately following surgery interfered with postoperative adhesion formation by promoting normal peristaltic movements in a rat model. Some components of the IFRD have important effects on improving the peristalsis and movement of gastrointestinal smooth muscle. Rhei Radix et Rhizoma (Daihuang), Aucklandiae Radix (Muxiang), and Cannabis Fructus (Huomaren) have been used extensively for treating gastrointestinal motor dysfunction [42, 43]. Emodin, an anthraquinone derivative of Rhei Radix et Rhizoma (Daihuang), has also been reported to possess an anti-inflammatory effect [44]. Furthermore, Aurantii Fructus (Zhiqiao) and Magnoliae Officinalis Cortex (Houpu) used together can synergistically increase gut motor function by involving muscarinic receptors and secondarily alpha-receptors [45]. Therefore, the IFRD is applied to promote the recovery of dynamic intestinal function and shorten the contact time with impaired peritoneum lining, thus preventing intra-abdominal adhesion in rats.

The present study has some limitations that should be noted and further explored to elucidate precise mechanisms. Further studies are required. In a future study, we will try to elucidate these mechanisms and identify which constituents or pure compounds serve as active ingredients for adhesion prevention in the IFRD.

## 5. Conclusion

**Figure 6:**
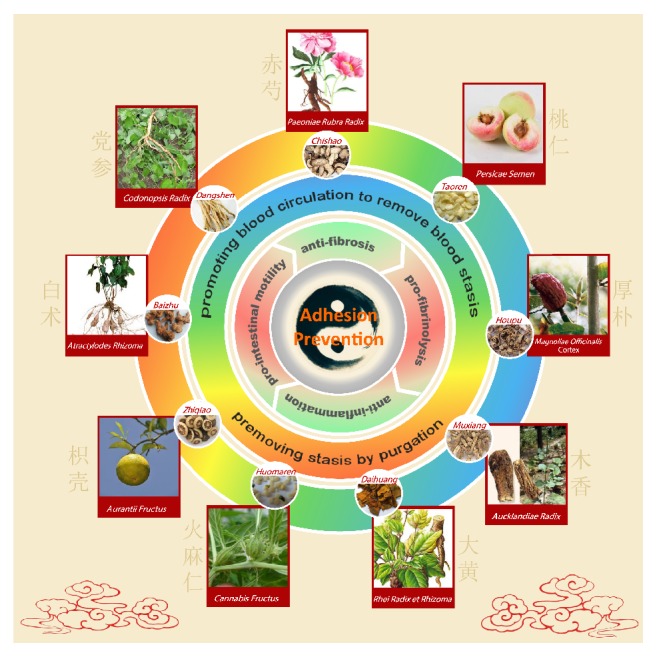
Preventative effects of the intestine function recovery decoction, a traditional Chinese medicine, on postoperative intra-abdominal adhesion formation in a rat model. The outermost circle illustrates the nine traditional Chinese medicinal (TCM) herbs, which are components of the intestine function recovery decoction (IFRD). The second circle shows that the IFRD possesses two types of TCM pharmacology, namely, “removing stasis by purgation” and “promoting blood circulation to remove blood stasis.” The third circle illustrates four types of underlying pharmacological action of the IFRD for preventing adhesive formation, including anti-inflammation, antifibrosis, profibrinolysis, and prointestinal motility. The kernel shows that the IFRD can effectively prevent adhesive formation independent of complementary TCM.

Our study found that the IFRD effectively prevented the formation of adhesions by decreasing inflammatory infiltration of the injured peritoneum and by reducing collagen deposition and fibrosis (Figure 6). This study demonstrated the wide-ranging potential value of the IFRD, a traditional Chinese medicine, in preventing postoperative adhesions, which could improve patient quality of life.

## Figures and Tables

**Table 1:** Constituents of intestine function recovery decoction.

Chinese name	Latin name	Family	Plant part	Dry weight (g)
Dangshen	*Codonopsis pilosula *Franch.	Campanulaceae	Radix	15
Baizhu	*Atractylodes macrocephala *Koidz.	Asteraceae	Rhizoma	15
Taoren	*Prunus persica *Batsch	Rosaceae	Semen	10
Chishao	*Paeonia lactiflora *Pall.	Ranunculaceae	Radix	15
Zhiqiao	*Citrus aurantium *L.	Rutaceae	Fructus	12
Houpu	*Magnolia officinalis *Rehd. et Wils.	Magnoliaceae	Cortex	15
Muxiang	*Aucklandia lappa *Decne.	Asteraceae	Radix	15
Huomaren	*Cannabis sativa *L.	Moraceae	Fructus	30
Daihuang	*Rheum palmatum *L.	Polygonaceae	Radix and Rhizoma	20

Total				147

**Table 2:** Steps of operation and postoperative intervention for each group.

Groups	Abdominal incision	Peritoneum abraded	Gastric infusion
Normal	Yes	No	No
Model	Yes	Yes	No
Control	Yes	Yes	10 mL/kg normal saline
Low dose IFRD	Yes	Yes	10 mL/kg IFRD (equivalent to 7.55 g of herbs/kg)
High dose IFRD	Yes	Yes	10 mL/kg IFRD (equivalent to 15.1 g of herbs/kg)

**Table 3:** The numerical scoring of adhesion described by Nair et al.

Grade	Criteria
0	No adhesion band is present
1	A single adhesion band forms between the viscera or between a viscus and the abdominal wall
2	Two bands form between the viscera or between the viscera and abdominal wall
3	More than two bands form between the viscera or between the viscera and abdominal wall, or the whole intestine forms a mass without adhering to the abdominal wall
4	The viscera have directly adhered to the abdominal wall, irrespective of the number of bands

**Table 4:** Effect of IFRD on rat abdominal adhesions after operation.

Group	*n*	Adhesions degree classification					Mean ± SD	Median adhesion score
		0	1	2	3	4		
Normal	8	7	1	0	0	0	0.125 ± 0.331	0
Model	8	0	0	1	3	4	3.375 ± 0.696	3.5
Control	8	0	0	2	4	2	3 ± 0.707	3
Low dose IFRD	8	1	2	3	2	0	1.75 ± 0.968	2
High dose IFRD	8	2	2	2	2	0	1.5 ± 1.118	1.5
*P* ^#^								<0.01

^#^ *Kruskal-Wallis analysis.*
